# Supplementary material for: OutbreakFlow: Model-based Bayesian inference of disease outbreak dynamics with invertible neural networks and its application to the COVID-19 pandemics in Germany
Source: PLoS Comput Biol. 2021 Oct 25;17(10):e1009472. doi: 10.1371/journal.pcbi.1009472 (PMC8584772; doi:10.1371/journal.pcbi.1009472)
Supplement: S1 Text — S1 Algorithm. Simulation-based offline learning. S2 Algorithm. Simulation-based online learning. S3 Algorithm. Simulation-based hybrid learning. S1 Table. Disease model priors. S2 Table. Intervention model priors. S3 Table. Observation model priors. S4 Table. Validation SIR model priors. S5 Table. Periodic SIR model priors. S1 Fig. Model-based predictions with non-identifiable parameters. S2 Fig. Calibration of OutbreakFlow trained with additional non-identifiable parameters. S3 Fig. Bivariate posteriors from real data. S4 Fig. Calibration of OutbreakFlow trained on the periodic SIR model. S5 Fig. Model-based predictions on 16 simulated time-series from the periodic SIR model. S6 Fig. Calibration of OutbreakFlow trained for inference on entire Germany. S7 Fig. Calibration of OutbreakFlow without a convolutional filtering network. S8 Fig. Model-based predictions using OutbreakFlow without a convolutional filtering network. S9 Fig. Calibration of OutbreakFlow without a recurrent summary network. S10 Fig. Model-based predictions using OutbreakFlow without a recurrent summary network. S11 Fig. Calibration of OutbreakFlow without a latent carrier compartment in the epidemiological model. S12 Fig. Model-based predictions using an epidemiological model without a carrier compartment. S13 Fig. Calibration of OutbreakFlow trained for inference on the German federal states. S14 Fig. Model predictions of new cases for each German federal state. S15 Fig. Model predictions of cumulative Covid-19 deaths (derived from predicted new cases) for each German federal state. S16 Fig. Marginal parameter posteriors from data available for the German federal state Baden-Württemberg. S17 Fig. Marginal parameter posteriors from data available for the German federal state Bavaria. S18 Fig. Marginal parameter posteriors from data available for the German federal state Berlin. S19 Fig. Marginal parameter posteriors from data available for the German federal state Brandenburg. S20 Fig. Marginal p [file pcbi.1009472.s001.pdf]

OutbreakFlow: Model-based Bayesian inference of disease outbreak dynamics with invertible neural networks and its application to the COVID-19 pandemics in Germany

Supporting Information

Stefan T. Radev[1], Frederik Graw[2], Simiao Chen[3,4], Nico T. Mutters[5], Vanessa M. Eichel[6], Till Bärnighausen[3,7,8], Ullrich Köthe[9*],

1 Institute of Psychology, Heidelberg University, Heidelberg, Germany
2 BioQuant - Center for Quantitative Biology, Heidelberg University, Heidelberg, Germany
3 Heidelberg Institute of Global Health, Heidelberg, Germany
4 Chinese Academy of Medical Sciences and Peking Union Medical College, Beijing, China
5 Institute for Hygiene and Public Health, University Hospital Bonn, Bonn, Germany
6 Center of Infectious Diseases, University Hospital Heidelberg, Heidelberg, Germany
7 Department of Global Health and Population, Harvard University, Harvard, Boston, USA
8 Africa Health Research Institute, Durban, South Africa
9 Computer Vision and Learning Lab, Heidelberg University, Heidelberg, Germany

1) Training Modes for OutbreakFlow (Algorithms S1-S3)

Offline Learning

Algorithm S1 OutbreakFlow training phase using offline learning

Require: f - invertible inference network, h - recurrent summary network, g - convolutional filtering network, ϕ - neural network parameters, S - total number of simulations, B - number of simulations per batch (batch size).

```
Generate a large reference table D^(S) := {θ^(s), x^(s)_{1:T}}_{s=1}^S by running the simulator S times.
repeat
    Sample a mini-batch of simulations: D^(B) ~ D^(S).
    Pass each simulated time-series through the filtering network: x̃^(b)_{1:T} = g(x^(b)_{1:T}).
    Pass the filtered time-series through the summary network: y^(b) = h(x̃_{1:T}).
    Pass each pair (θ^(b), y^(b)) through the inference network: z^(b) = f(θ^(b), y^(b)).
    Compute loss from batch: L(φ) = −Σ_{b=1}^B log q_φ(θ^(b) | x^(b)_{1:T}).
    Update neural network parameters φ via backpropagation.
until convergence to φ*
Return trained networks g, h, f with parameters φ*.
```

Online Learning

Algorithm S2 OutbreakFlow training phase using online learning

Require: f - invertible inference network, h - recurrent summary network, g - convolutional filtering network, ϕ - neural network parameters, B - number of simulations per iteration (batch size).

repeat
    Generate a mini-batch $\mathcal{D}^{(B)} := \{\theta^{(b)}, x_{1:T}^{(b)}\}_{b=1}^{B}$ by running the simulator B times.
    Pass each simulated time-series through the filtering network: $\widetilde{x}_{1:T}^{(b)} = g(x_{1:T}^{(b)})$.
    Pass the filtered time-series through the summary network: $y^{(b)} = h(\widetilde{x}_{1:T})$.
    Pass each pair $(\theta^{(b)}, y^{(b)})$ through the inference network: $z^{(b)} = f(\theta^{(b)}, y^{(b)})$.
    Compute loss from batch: $\mathcal{L}(\phi) = -\sum_{b=1}^{B} \log q_\phi(\theta^{(b)} \mid x_{1:T}^{(b)})$.
    Update neural network parameters ϕ via backpropagation.
until convergence to ϕ^*
Return trained networks g, h, f with parameters ϕ^*.

Round-Based Learning

Algorithm S3 OutbreakFlow training phase using round-based hybrid learning

Require: f - invertible inference network, h - recurrent summary network, g - convolutional filtering network, ϕ - neural network parameters, R - number of rounds, S - number of simulations per round, B - batch size.

Initialize reference table $\mathcal{D}^{(R\times S)} := \{\}$.
for $r = 1, ..., R$ **do**
    Generate synthetic data $\mathcal{D}_r^{(S)} := \{\theta^{(s)}, x_{1:T}^{(s)}\}_{s=1}^{S}$ by running the simulator S times.
    Aggregate data: $\mathcal{D}^{(R\times S)} := \mathcal{D}^{(R\times S)} \cup \mathcal{D}_r^{(S)}$.
    repeat
        Sample a mini-batch of simulations: $\mathcal{D}^{(B)} \sim \mathcal{D}^{(R\times S)}$.
        Pass each simulated time-series through the filtering network: $\widetilde{x}_{1:T}^{(b)} = g(x_{1:T}^{(b)})$.
        Pass the filtered time-series through the summary network: $y^{(b)} = h(\widetilde{x}_{1:T})$.
        Pass each pair $(\theta^{(b)}, y^{(b)})$ through the inference network: $z^{(b)} = f(\theta^{(b)}, y^{(b)})$.
        Compute loss from batch: $\mathcal{L}(\phi_r) = -\sum_{b=1}^{B} \log q_{\phi_r}(\theta^{(b)} \mid x_{1:T}^{(b)})$.
        Update neural network parameters ϕ via backpropagation.
    until convergence to ϕ_r^*
end for
Return trained networks g, h, f with parameters ϕ_R^*.

2) Parameter Priors for the Model of the Early Covid-19 Pandemics in Germany (Tables S1-S3)

Disease Model

Table S1. Description of disease model parameters and corresponding prior distributions

Parameter	Symbol	Prior Distribution
Number of initially exposed individuals	E_0	$\text{Gamma}(2, 30)$
Risk of infection from symptomatic patients	β	$\text{LogNormal}(\log(0.25), 0.3)$
Rate at which exposed cases become infectious	γ	$\text{LogNormal}(\log(1/6.5), 0.5)$
Rate at which symptoms manifest	η	$\text{LogNormal}(\log(1/3.2), 0.5)$
Rate at which symptomatic individuals recover	μ	$\text{LogNormal}(\log(1/8), 0.2)$
Rate at which undiagnosed individuals recover	θ	$\text{Uniform}(1/14, 1/3)$
Rate at which critical cases die	d	$\text{Uniform}(1/14, 1/3)$
Probability of remaining undetected/undiagnosed	α	$\text{Uniform}(0.005, 0.99)$
Probability of dying from the disease	δ	$\text{Uniform}(0.01, 0.3)$

Intervention Model

Table S2. Description of intervention model parameters controlling the time-varying transmission rate

Parameter	Symbol	Prior Distribution
Onset date of each change to take effect	t_1	$\text{Normal}(2020/03/09 = \text{Day } 8, 3)$
	t_2	$\text{Normal}(2020/03/16 = \text{Day } 15, 3)$
	t_3	$\text{Normal}(2020/03/23 = \text{Day } 22, 3)$
	t_4	$\text{Normal}(2020/05/06 = \text{Day } 66, 3)$
Duration of each change to fully manifest itself	Δt_j	$\text{LogNormal}(\log(3), 0.3)$
Transmission rates before / after each change	λ_0	$\text{LogNormal}(\log(1.2), 0.5)$
	λ_1	$\text{LogNormal}(\log(0.6), 0.5)$
	λ_2	$\text{LogNormal}(\log(0.3), 0.5)$
	λ_3	$\text{LogNormal}(\log(0.1), 0.5)$
	λ_4	$\text{LogNormal}(\log(0.15), 0.5)$

Observation Model

Table S3. Description of observation model parameters controlling reporting properties

Parameter	Symbol	Prior Distribution
Reporting delays (lags)	$L_{\mathcal{C} \in \{I,R,D\}}$	$\text{LogNormal}(\log(8), 0.2)$
Weekly modulation amplitudes	$A_{\mathcal{C} \in \{I,R,D\}}$	$\text{Beta}(0.7, 0.17)$
Weekly modulation phases	$\Phi_{\mathcal{C} \in \{I,R,D\}}$	$\text{VonMises}(0.01)$
Reporting noise scale	$\sigma_{\mathcal{C} \in \{I,R,D\}}$	$\text{Gamma}(1, 5)$

3) Testing and Validation of OutbreakFlow (Table S4, Figures S1-S3)

Parameter Priors

Table S4. Description of model parameters and corresponding prior distributions

Parameter	Symbol	Prior Distribution
Initial transmission rate	λ	$\text{LogNormal}(\log(0.4), 0.5)$
Recovery rate of infected individuals	μ	$\text{LogNormal}(\log(1/8), 0.2)$
Reporting delay (lag)	L	$\text{LogNormal}(\log(8), 0.2)$
Number of initially infected individuals	I_0	$\text{Gamma}(2, 20)$
Dispersion of the negative binomial distribution	ψ	$\text{Exponential}(5)$

Results with Uninformative Parameters

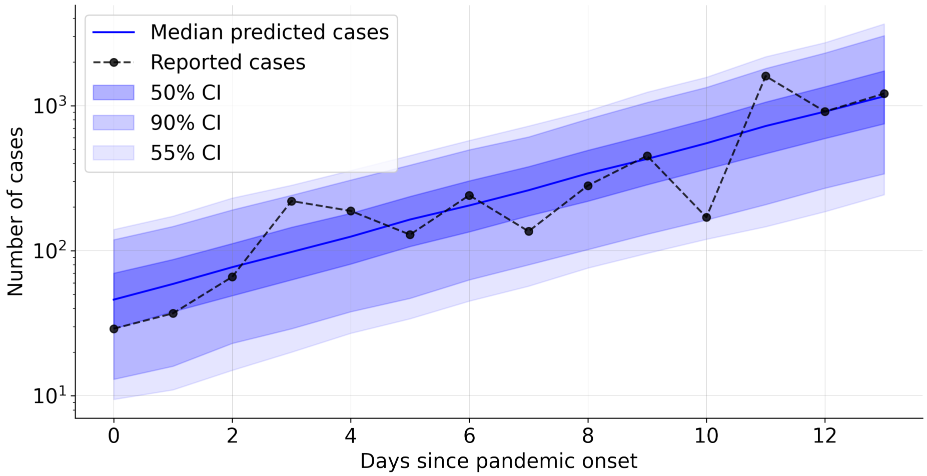

Fig S1. Posterior predictions on new cases from the first 2 weeks of the Covid-19 pandemics in Germany. We observe accurate reproduction of the real data and well-calibrated uncertainty despite the addition of uninformative parameters.

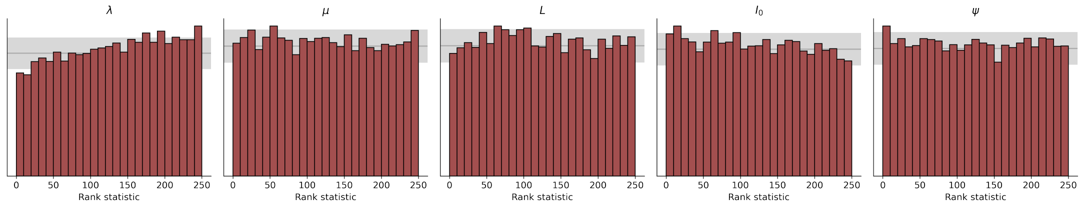

Fig S2. Simulation-based probabilistic calibration of the marginal approximate posteriors obtained by the network trained to estimate the simple SIR-model. The addition of uninformative parameters does not seem to induce systematic biases across the marginal posteriors.

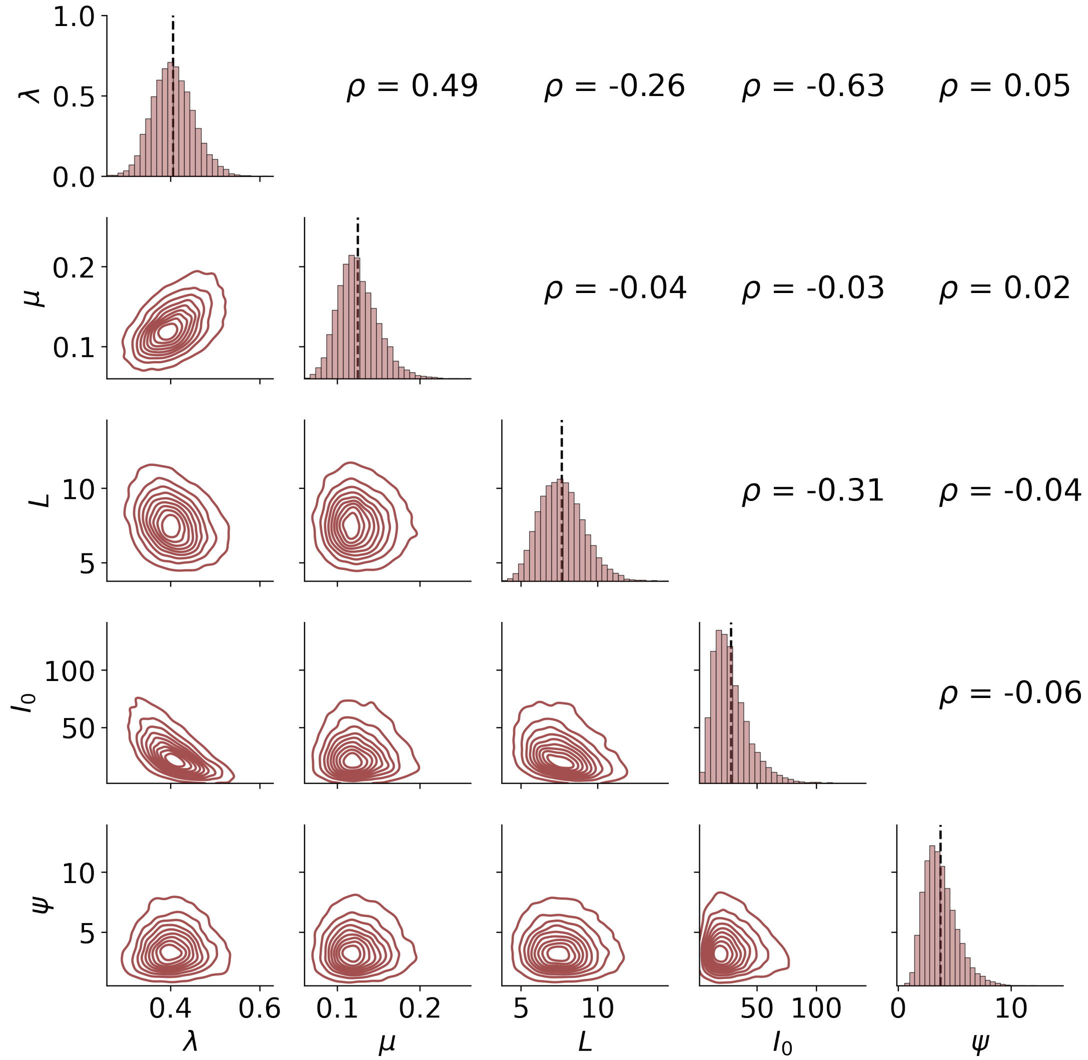

Fig S3. Bivariate and univariate posteriors obtained by applying the simple SIR model (Eq. 16-18) to cases reported in the first 2 weeks of the Covid-19 pandemic in Germany. The addition of uninformative parameters does not lead to results which diverge from or lead to different conclusions from the main analysis (see Fig 3). Dashed black lines on the diagonal indicate posterior means.

4) Periodic SIR Model (Table S5, Figures S4-S5)

In addition to the analyses in the main paper, we explored the behavior of OutbreakFlow on a noisy periodic SIR model with potentially chaotic behavior. Following [1], the model is defined by the following set of ordinary differential equations (ODEs):

$$\frac{dS}{dt} = -\beta(t)\left(\frac{S\,I}{N}\right) + \mu\,N - \mu\,S \tag{S1}$$

$$\frac{dI}{dt} = \beta(t)\left(\frac{S\,I}{N}\right) - (\gamma + \mu)\,I \tag{S2}$$

$$\frac{dR}{dt} = \gamma\,I - \mu\,R \tag{S3}$$

The model assumes that at time t, the population is composed of S susceptible, I infected, and R removed (recovered or immune) individuals. Vital dynamics are taken into account by considering a natural mortality rate per capita $\mu > 0$ and a number μN of births per time unit, where N is the total population size. Since we assume that the disease is non-lethal, the size of the population is constant $N = S + I + R$. We further assume a transmission rate $\beta(t)$ which is a periodic function of time with a fixed period T. Infected individuals recover at a rate $\gamma > 0$.

The transmission rate $\beta(t)$ varies as a function of time according to:

$$\beta(t) = \beta_0(1 + \epsilon\,\cos(2\pi\omega t)) \tag{S4}$$

Further, we assume the following observation model for the observed new infections I_{obs}:

$$I_t^{(obs)} \sim \text{Normal}(I_t, \sqrt{I_t}\sigma), \tag{S5}$$

Thus, the full parameter vector to be estimated is $\theta = (\beta_0, \mu, \gamma, \epsilon, \omega, \sigma)$. Prior distributions for the parameters are given in the below table.

Table S5. Description of model parameters and corresponding prior distributions

Parameter	Symbol	Prior Distribution
Base transmission rate	β_0	Uniform$(1, 3)$
Natural mortality rate	μ	Uniform$(1/14, 1/3)$
Recovery rate	γ	Uniform$(1/13, 1/3)$
Seasonal modulation amplitude	ϵ	Uniform$(1/10, 1)$
Seasonal modulation frequency	ω	Uniform$(0.9, 10)$
Noise factor of the observation model	σ	Exponential(5)

In order to calibration, estimation, and predictions, we train the OutbreakFlow networks via online learning for a total of 50000 mini-batch simulations from the model.

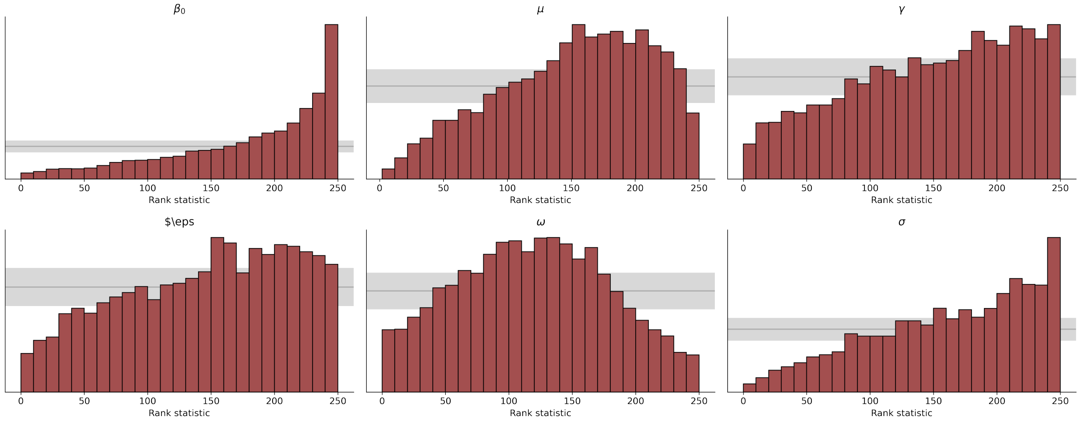

Fig S4. Simulation-based probabilistic calibration of the marginal approximate posteriors obtained by OutbreakFlow trained on the seasonal SIR model. The results indicate poor calibration, since notable deviations from uniformity are present, revealing systematic parameter recovery and uncertainty quantification deficiencies.

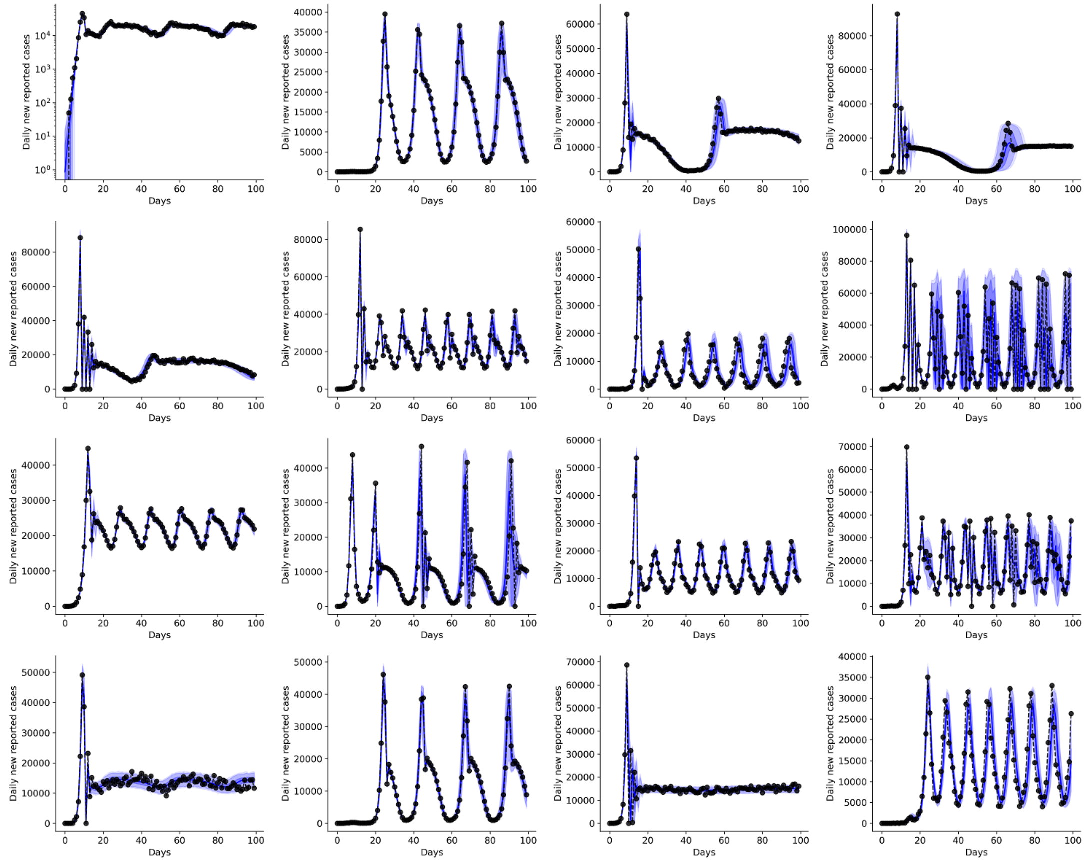

Fig S5. Model-based predictions on 16 simulated time-series from the periodic SIR model. Despite poor calibration, the network is able to encode the periodic structure of the data well and appears to capture the average dynamics.

5) Simulation-Based Calibration of OutbreakFlow for Inference on Entire Germany (Figure S6)

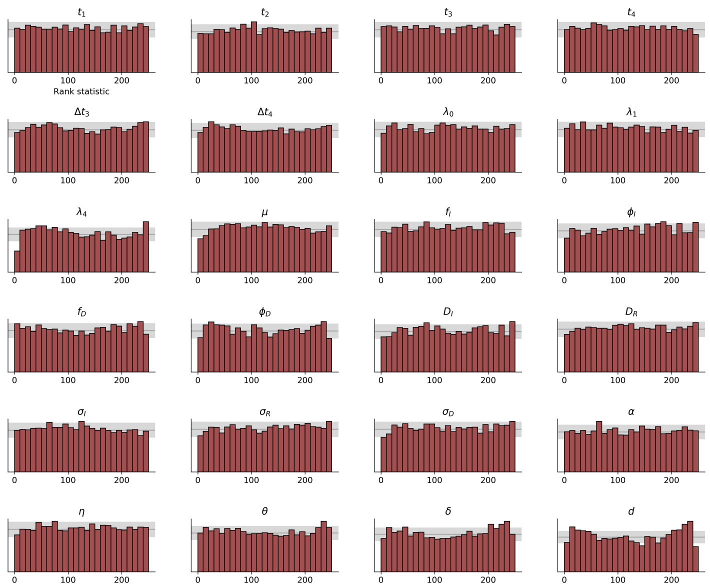

Fig S6. Simulation-based probabilistic calibration of the marginal approximate posteriors obtained by the network trained for inference on entire Germany. Uniformly distributed histograms of the rank statistic indicate no systematic biases in the estimation of location and scale of the true marginal posteriors.

6) Ablation Study Results (Figures S7-S12)

6.1) Removing the Convolutional Filtering Network

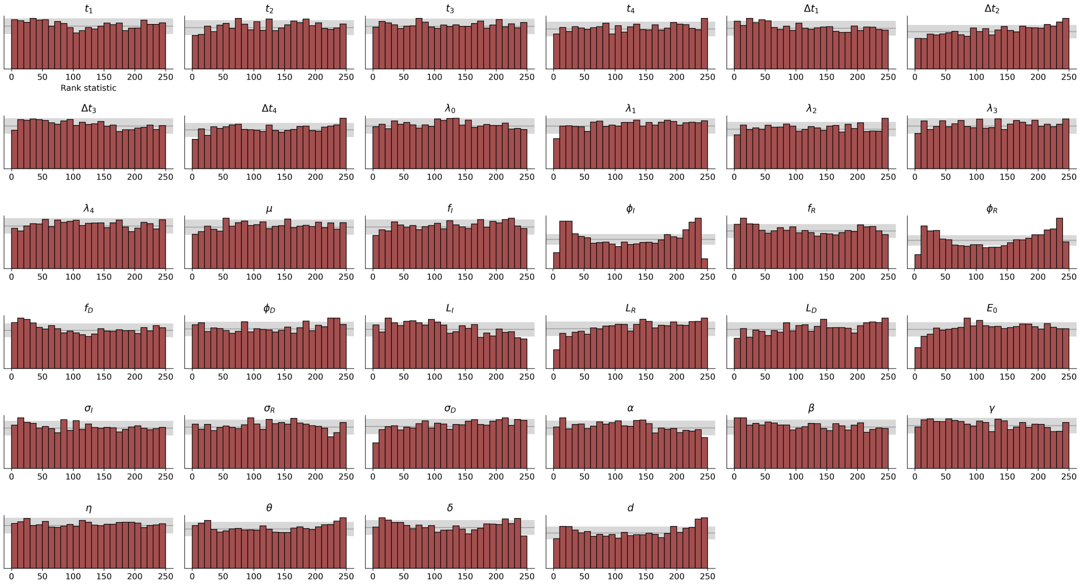

Fig S7. Simulation-based probabilistic calibration of the marginal approximate posteriors obtained by OutbreakFlow without a convolutional filtering network. Uniformly distributed histograms of the rank statistic indicate no systematic biases in the estimation of location and scale of the true marginal posteriors. We observe slightly worse calibration, especially regarding the observation model parameters, when using this reduced architecture.

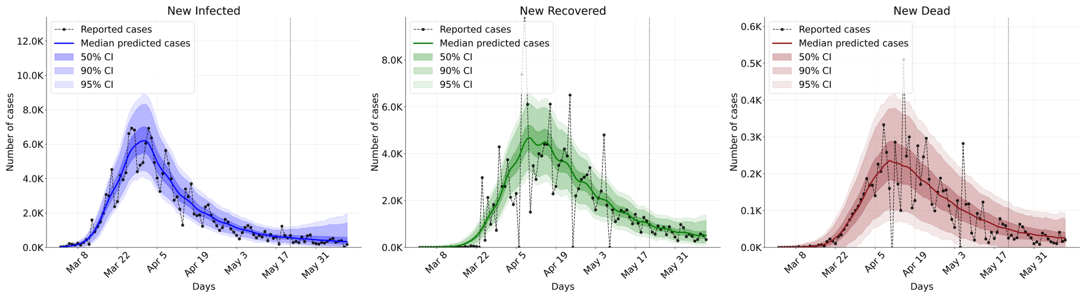

Fig S8. Posterior predictions and forecasts of new cases based on the same data as in the main text but using an OutbreakFlow architecture without a convolutional filtering network. We observe that this reduced architecture has difficulties recovering the weekly modulation. Cases to the left of the vertical dashed line were used for posterior checking (model training) and cases to the right for posterior forecasts (predictions) on unseen data.

6.2) Removing the Recurrent Summary Network

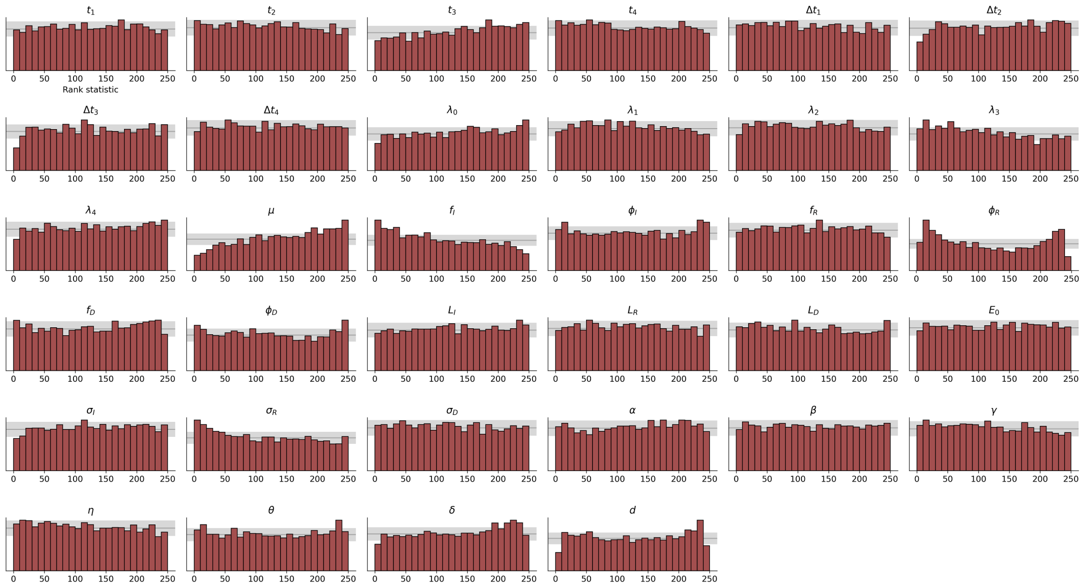

Fig S9. Simulation-based probabilistic calibration of the marginal approximate posteriors obtained by OutbreakFlow without a recurrent summary network. Uniformly distributed histograms of the rank statistic indicate no systematic biases in the estimation of location and scale of the true marginal posteriors. We observe slightly worse calibration, especially regarding the observation model parameters, when using this reduced architecture.

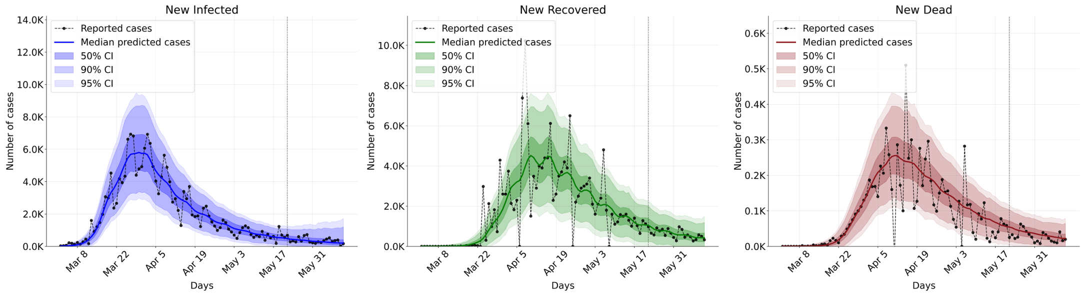

Fig S10. Posterior predictions and forecasts of new cases based on the same data as in the main text but using an OutbreakFlow architecture without a recurrent summary network. We observe that this reduced architecture has difficulties recovering the weekly modulation. Cases to the left of the vertical dashed line were used for posterior checking (model training) and cases to the right for posterior forecasts (predictions) on unseen data.

6.3) Removing the Observation Model

Inference on the observed epidemiological time series yielded only divergent simulations.

6.4) Removing the Intervention Model

Inference on the observed epidemiological time series yielded only divergent simulations.

6.5) Removing the Latent Carrier Compartment

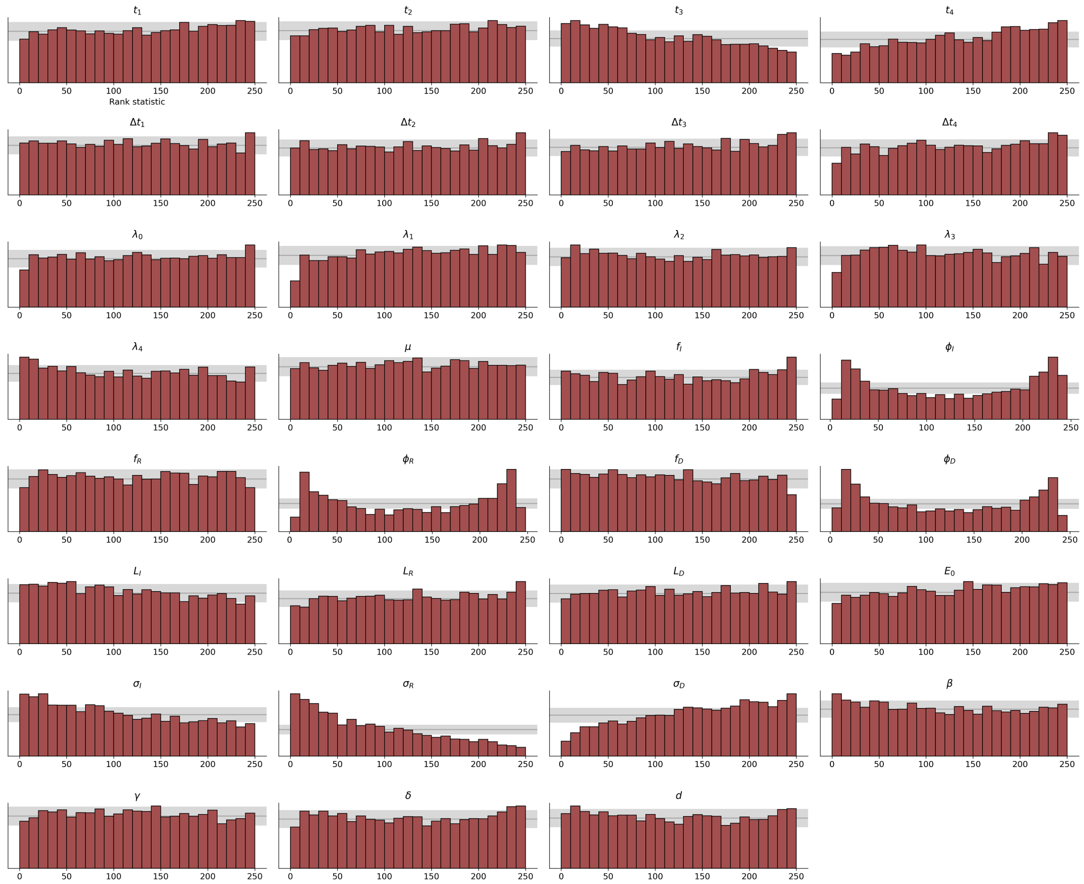

Fig S11. Simulation-based probabilistic calibration of the marginal approximate posteriors obtained by OutbreakFlow on a SEIRD model (lacking a latent carrier compartment). Uniformly distributed histograms of the rank statistic indicate no systematic biases in the estimation of location and scale of the true marginal posteriors. We observe slightly worse calibration, especially regarding the observation model parameters, when using this reduced model.

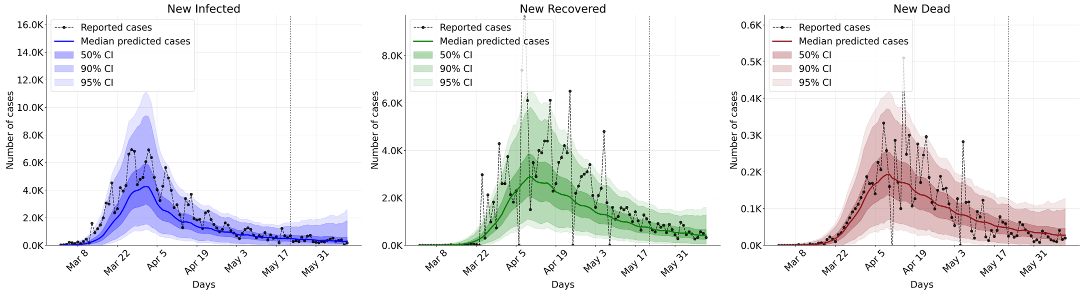

Fig S12. Posterior predictions and forecasts of new cases based on the same data as in the main text but using an OutbreakFlow architecture trained on a reduced SEIRD model (lacking a latent carrier compartment). We observe that this reduced model does not reproduce the data well and largely underestimates the reported cases, especially during the beginning and peak of the pandemic wave. Note: Cases to the left of the vertical dashed line were used for posterior checking (model training) and cases to the right for posterior forecasts (predictions) on unseen data.

7) Simulation-Based Calibration of OutbreakFlow for Inference on the Individual German States (Figure S13)

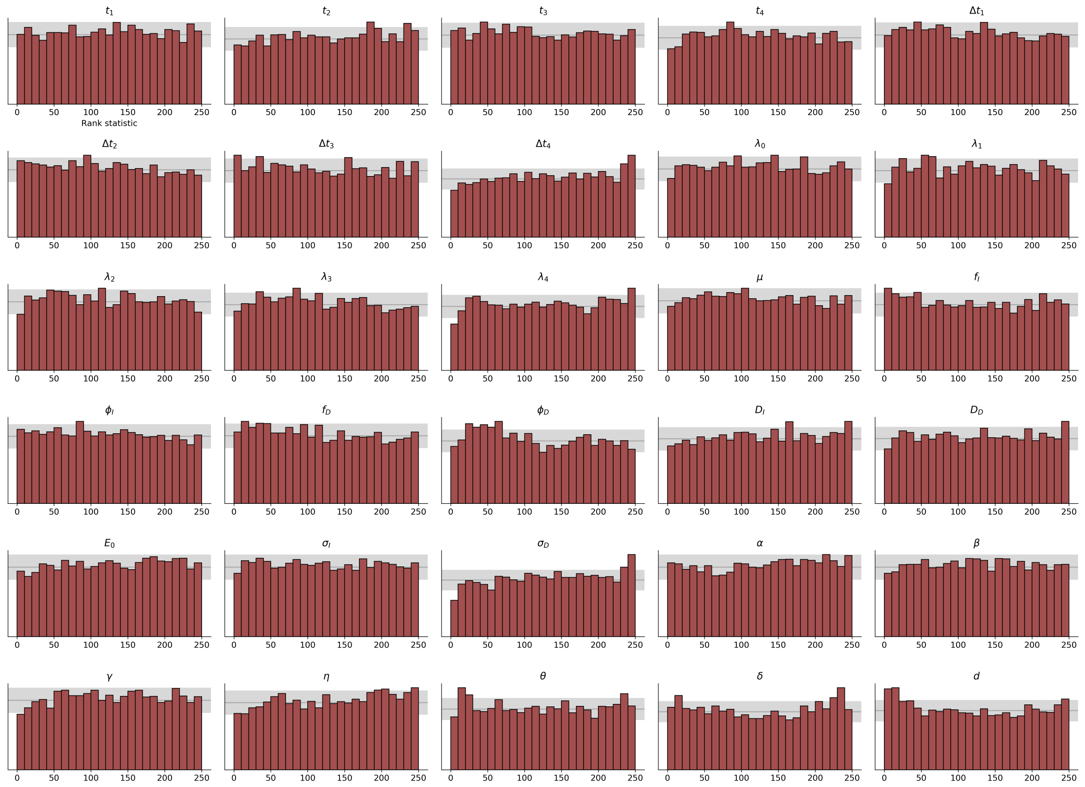

Fig S13. Simulation-based probabilistic calibration of the marginal approximate posteriors obtained by the networks trained for amortized inference on all German federal states. Uniformly distributed histograms of the rank statistic indicate no systematic biases in the estimation of location and scale of the true marginal posteriors.

8) Predicted New Cases per German Federal State (Figure S14)

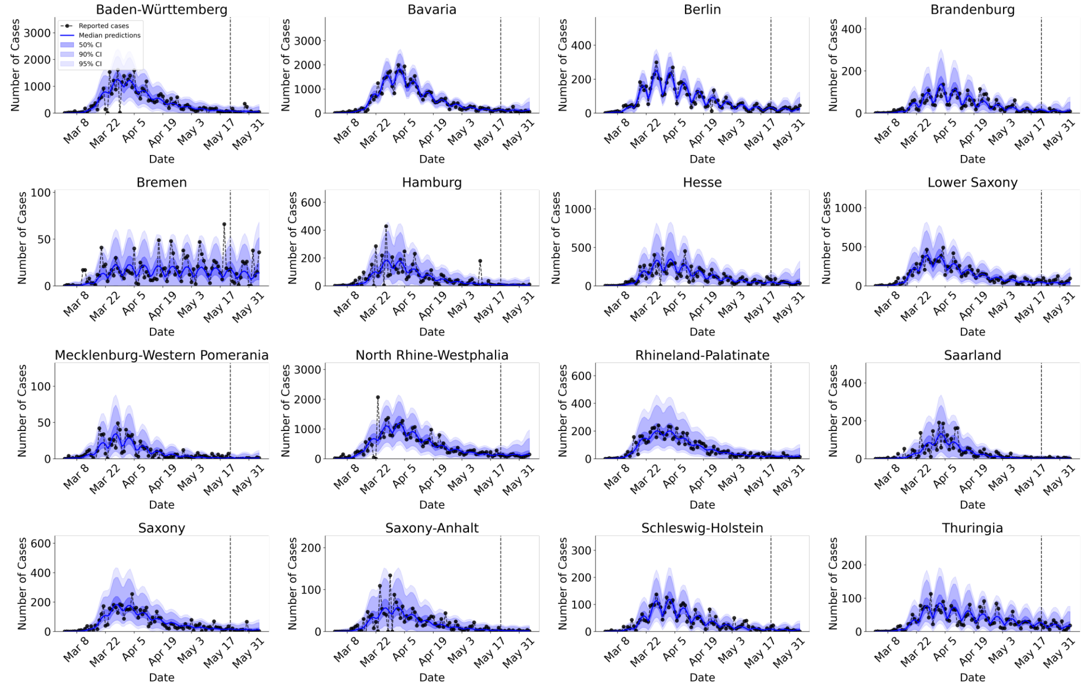

Fig S14. Model predictions of new cases Covid-19 deaths for each German federal state. Cases to the left of the vertical dashed line (8 weeks) were used for model fitting and posterior checking and cases to the right (3 weeks) for forecasts on new data. We observe good matches between the model's median predictions and past and future reported cases for each German federal state.

9) Predicted Cumulative Deaths per German Federal State (Figure S15)

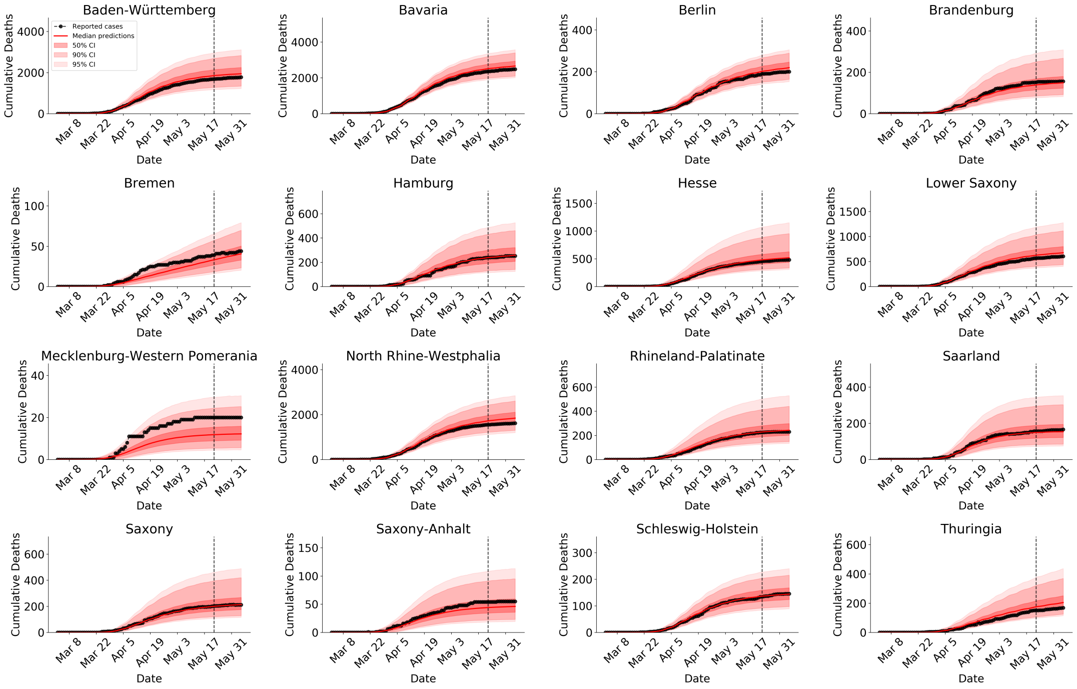

Fig S15. Model predictions of cumulative Covid-19 deaths (derived from predicted new cases) for each German federal state. Cases to the left of the vertical dashed line (8 weeks) were used for model fitting and posterior checking and cases to the right (3 weeks) for forecasts on new data. We observe good matches between the model's median predictions and past and future reported deaths for each German federal state. However, the number of deaths in the state Mecklenburg-Western Pomerania over time is underestimated (although it lies mostly within the estimated 95%-CI), which is probably due to the very low counts (lowest among all German federal states).

10) Marginal Parameter Posteriors per Federal State (Figures S16-S31)

Baden-Württemberg

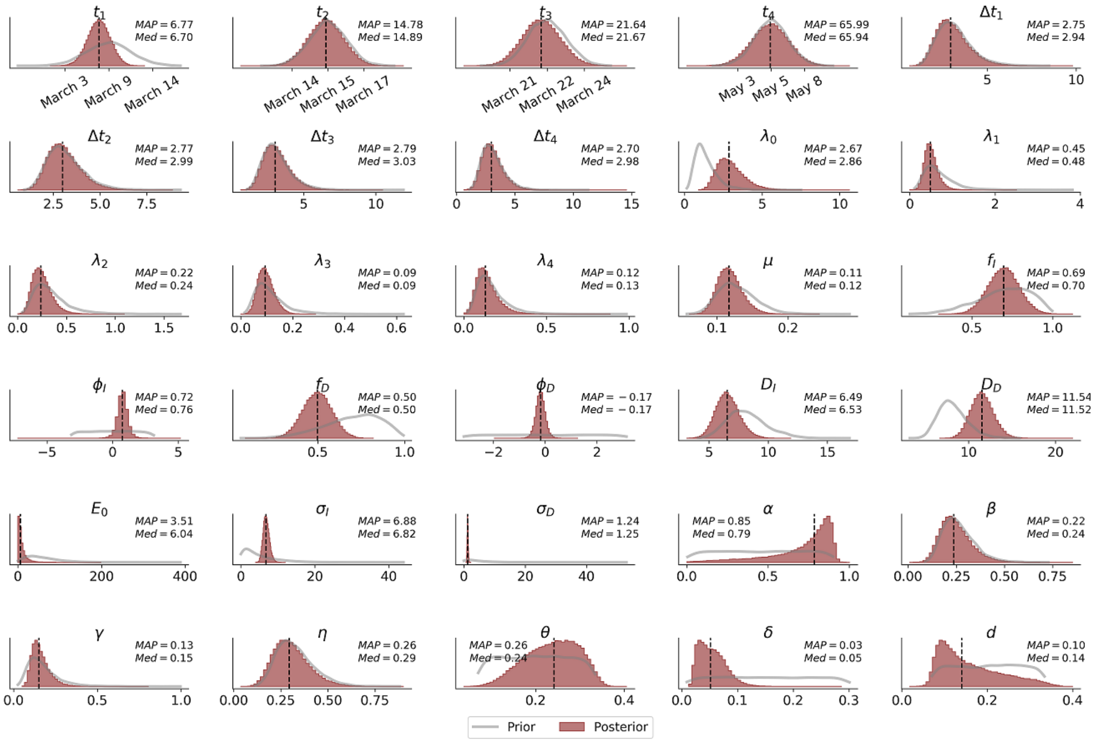

Fig S16. Marginal parameter posteriors from data available for the German federal state Baden-Württemberg.

Bavaria

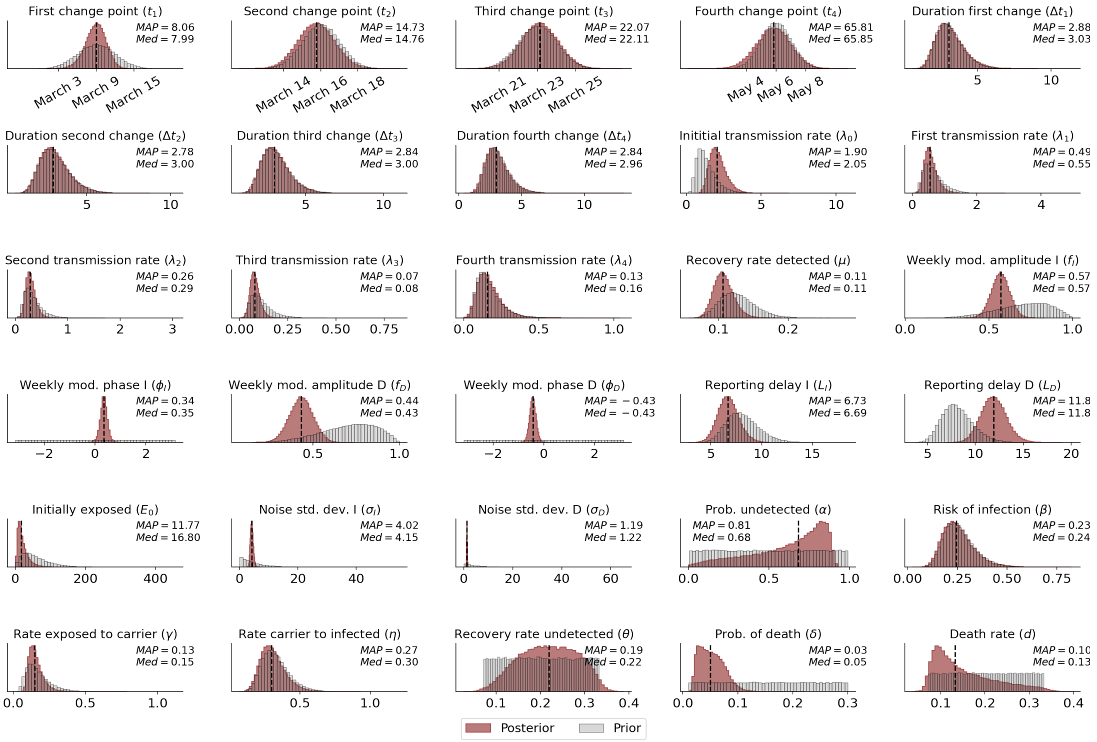

Fig S17. Marginal parameter posteriors from data available for the German federal state Bavaria.

Berlin

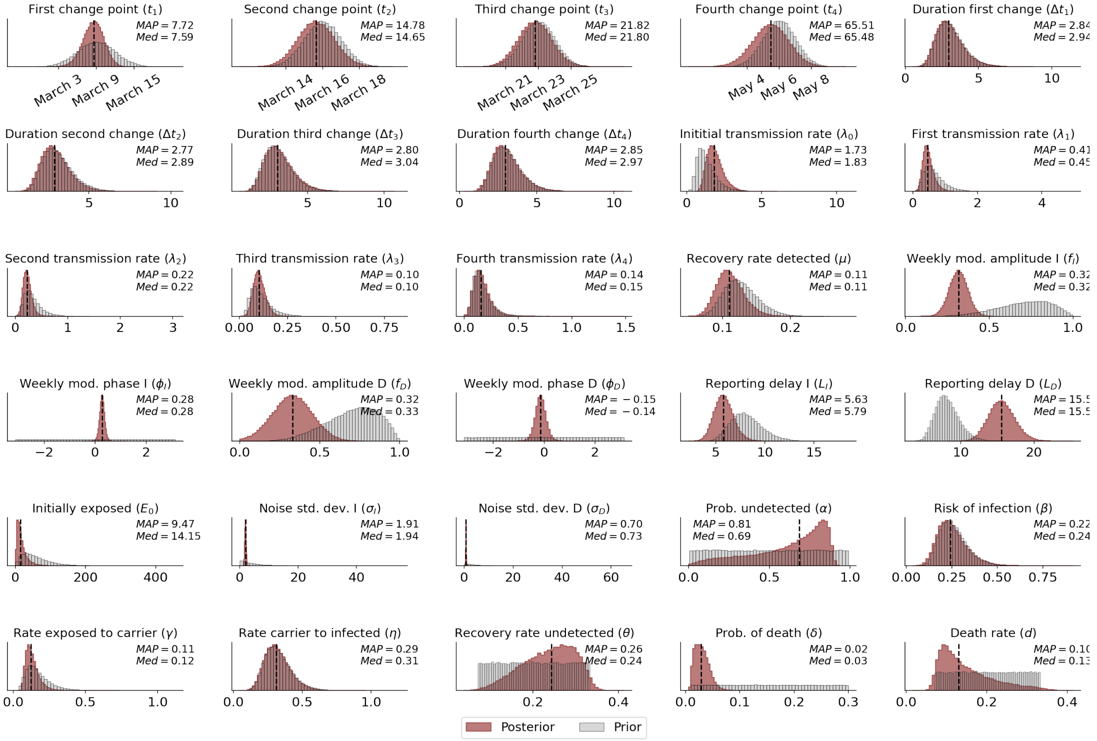

Fig S18. Marginal parameter posteriors from data available for the German federal state Berlin.

Brandenburg

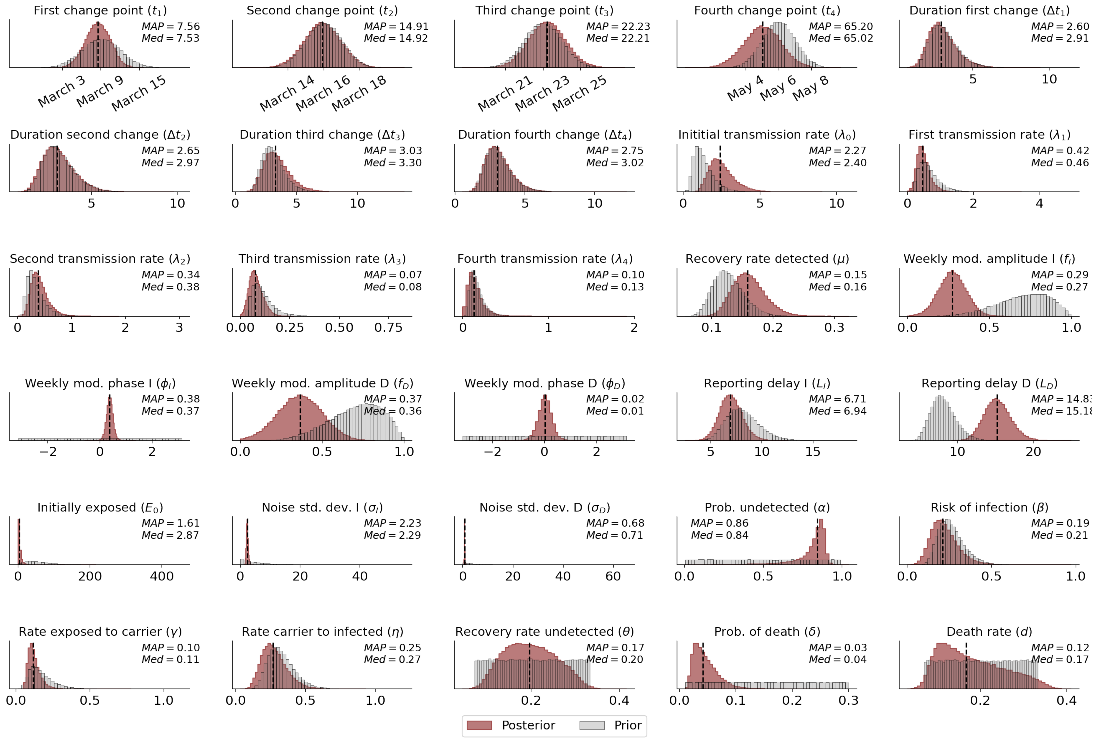

Fig S19. Marginal parameter posteriors from data available for the German federal state Brandenburg.

Bremen

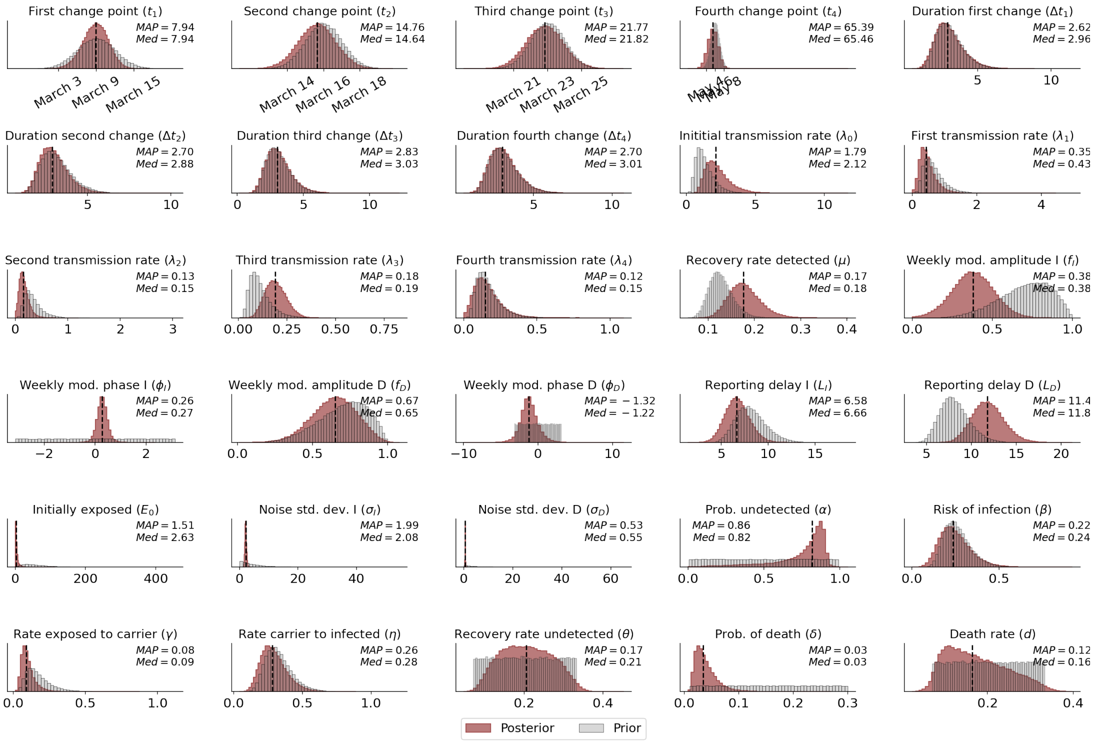

Fig S20. Marginal parameter posteriors from data available for the German federal state Bremen.

Hamburg

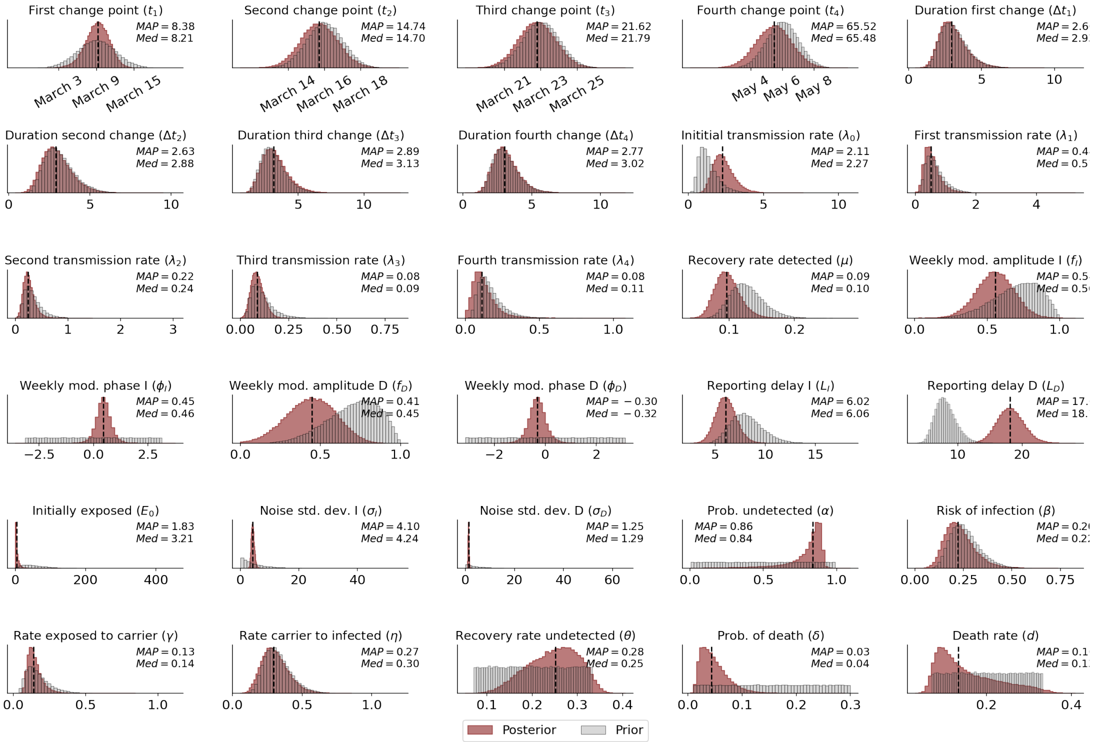

Fig S21. Marginal parameter posteriors from data available for the German federal state Hamburg.

Hesse

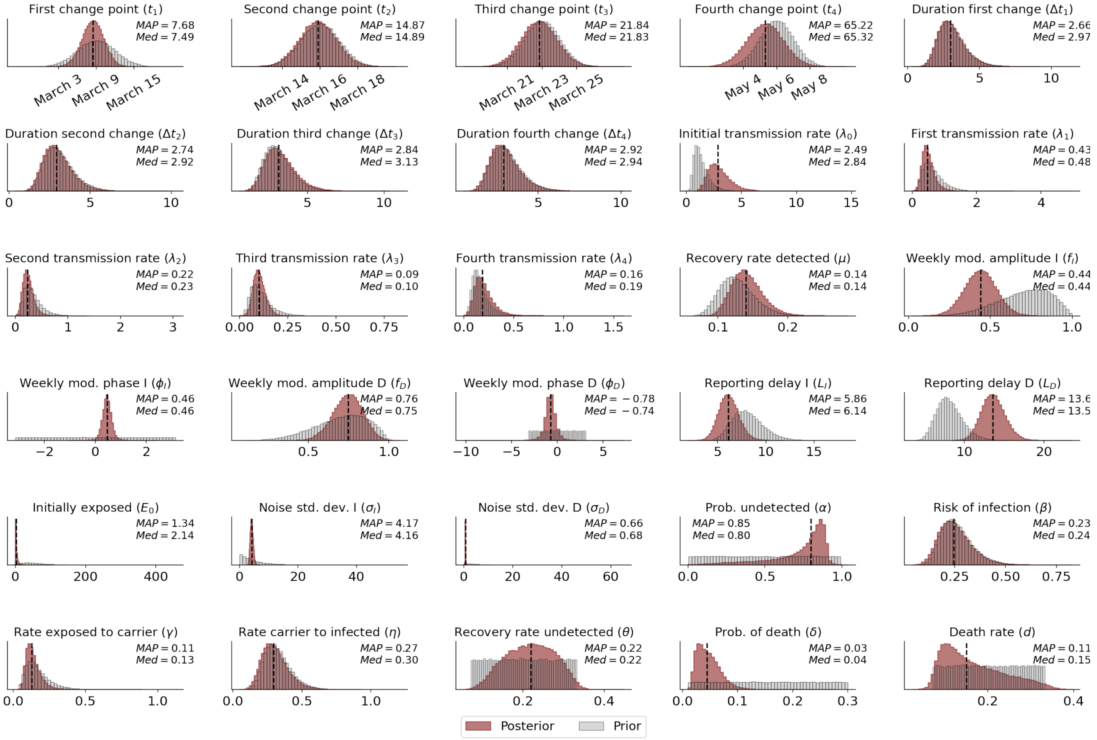

Fig S22. Marginal parameter posteriors from data available for the German federal state Hesse.

Saxony

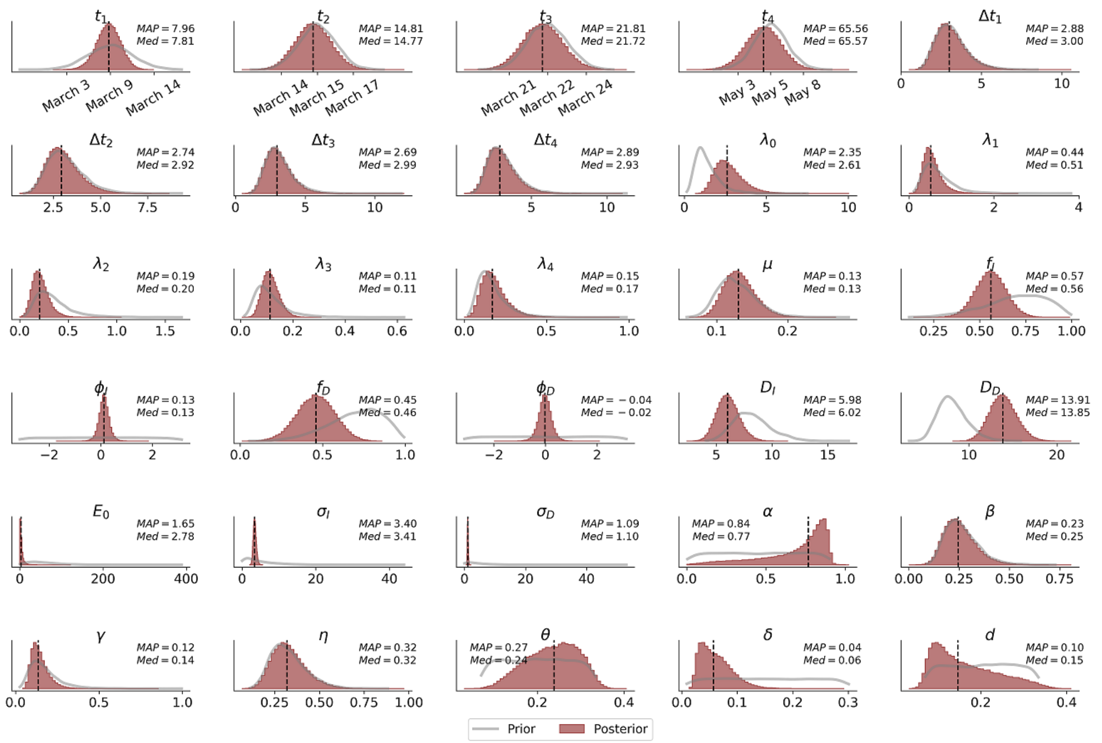

Fig S23. Marginal parameter posteriors from data available for the German federal state Lower Saxony.

Mecklenburg-Western Pomerania

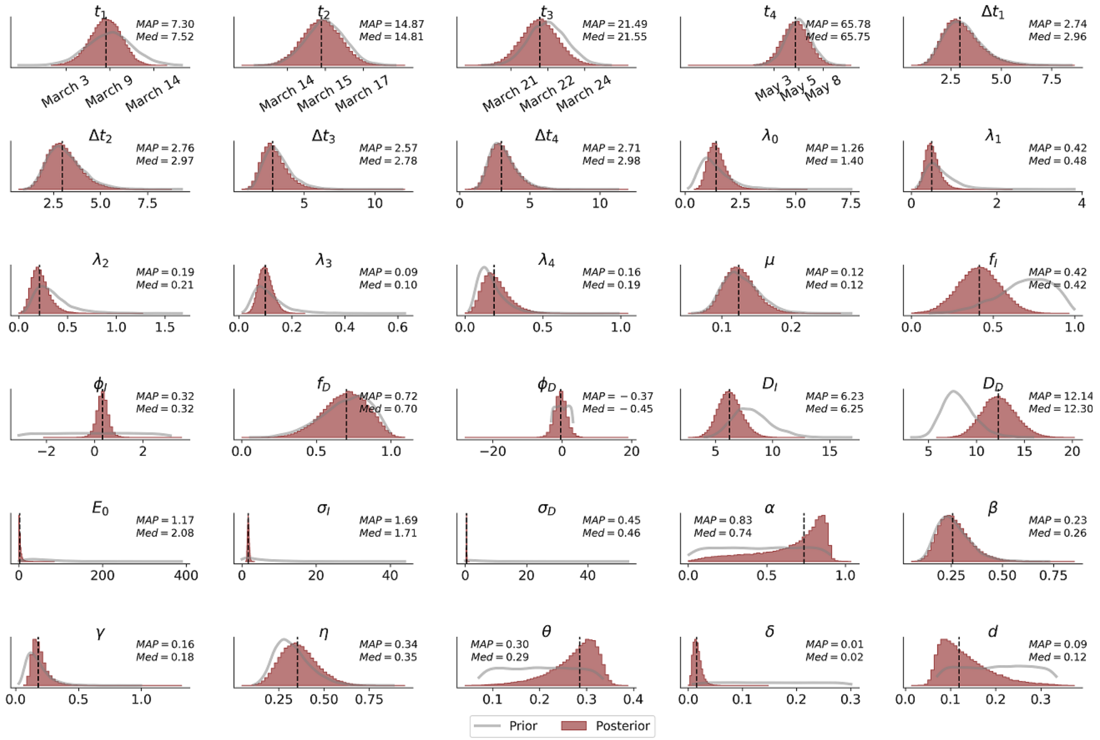

Fig S24. Marginal parameter posteriors from data available for the German federal state Mecklenburg-Western Pomerania.

North Rhine-Westphalia

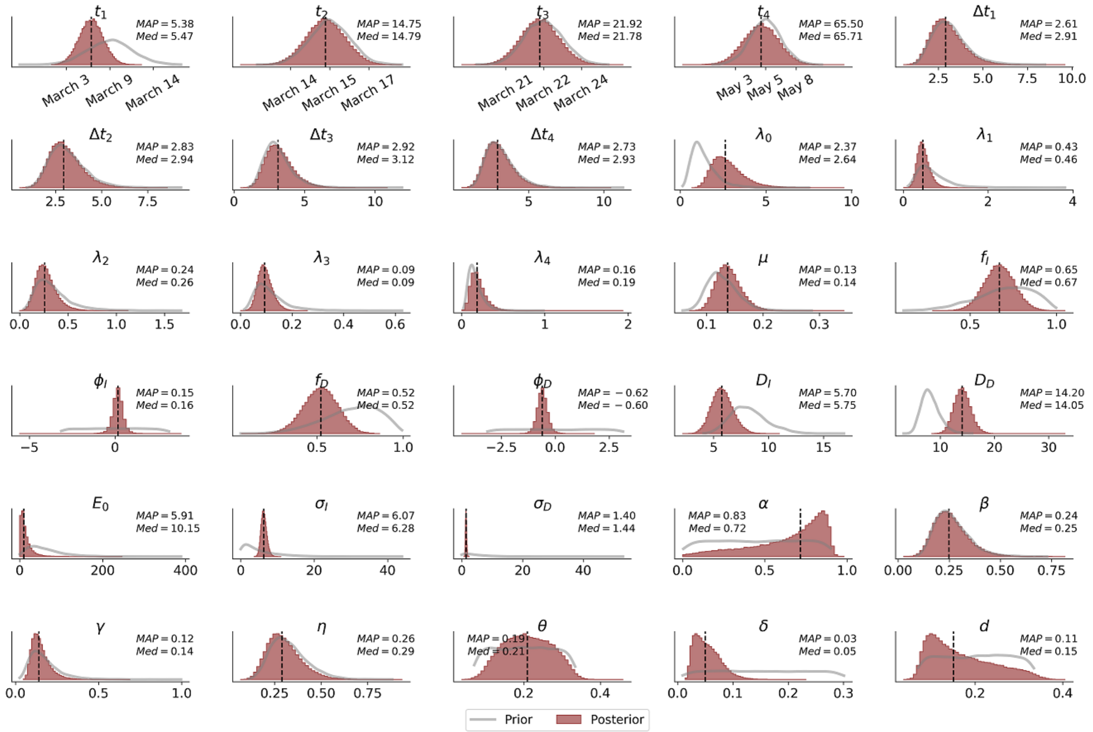

Fig S25. Marginal parameter posteriors from data available for the German federal state North Rhine-Westphalia.

Rhineland-Palatinate

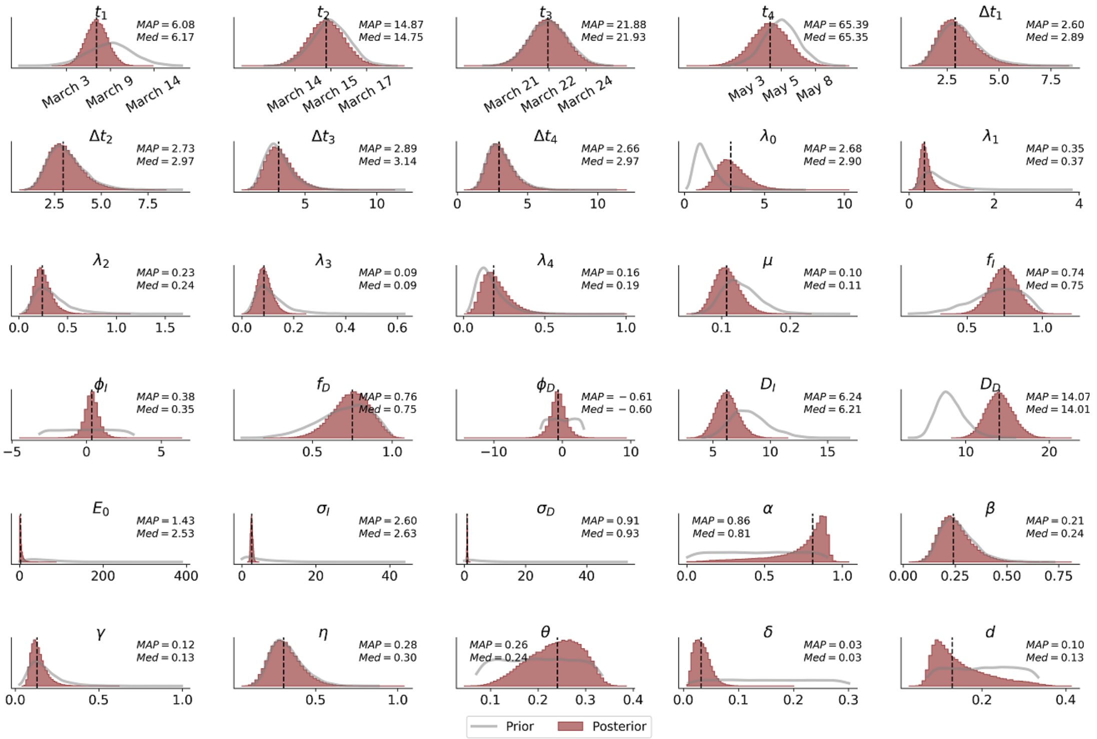

Fig S26. Marginal parameter posteriors from data available for the German federal state Rhineland-Palatinate.

Saarland

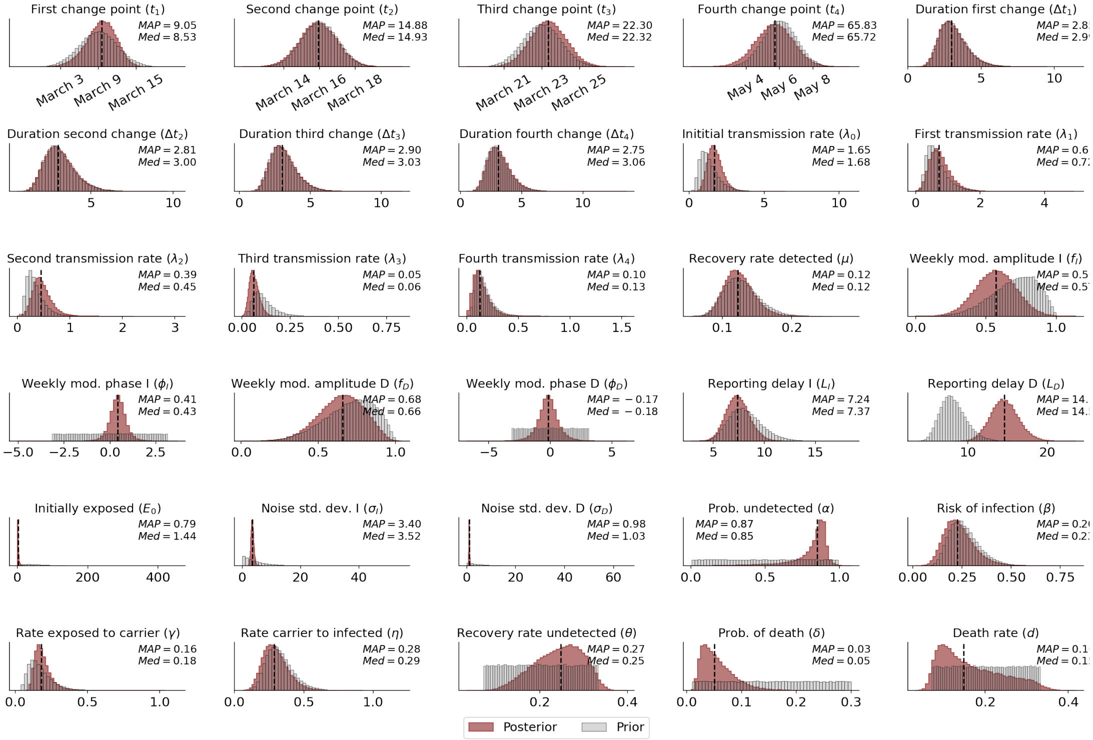

Fig S27. Marginal parameter posteriors from data available for the German federal state Saarland.

Saxony-Anhalt

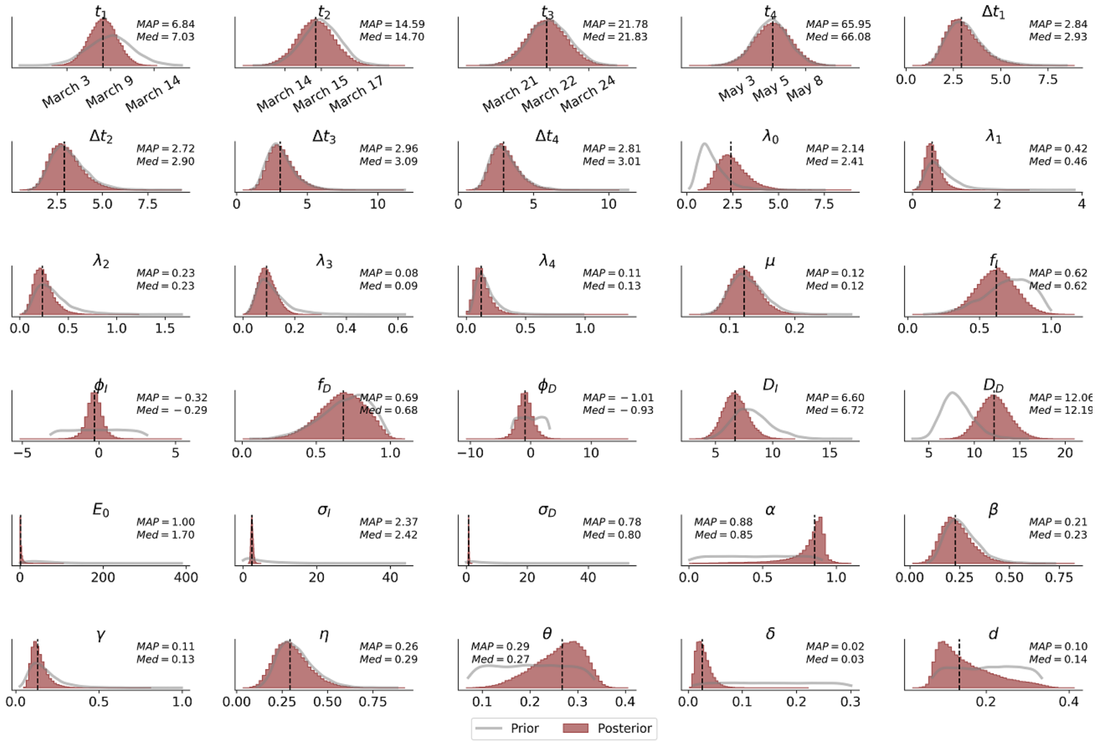

Fig S28. Marginal parameter posteriors from data available for the German federal state Saxony-Anhalt.

Saxony

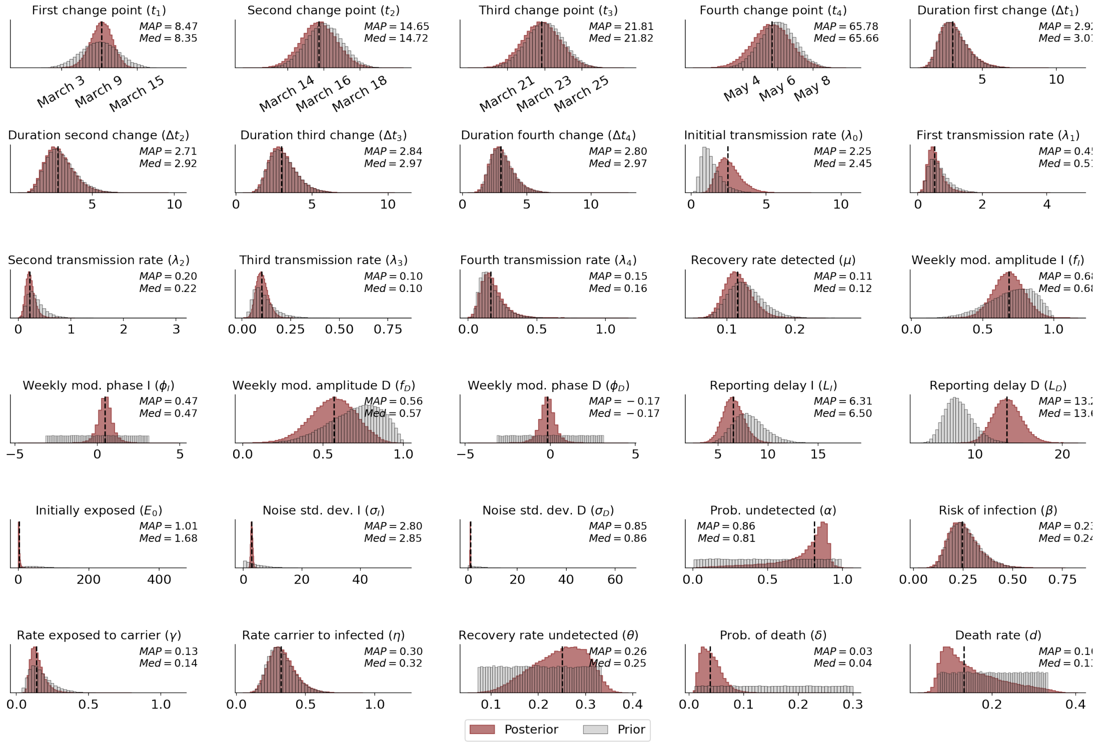

Fig S29. Marginal parameter posteriors from data available for the German federal state Saxony.

Schleswig-Holstein

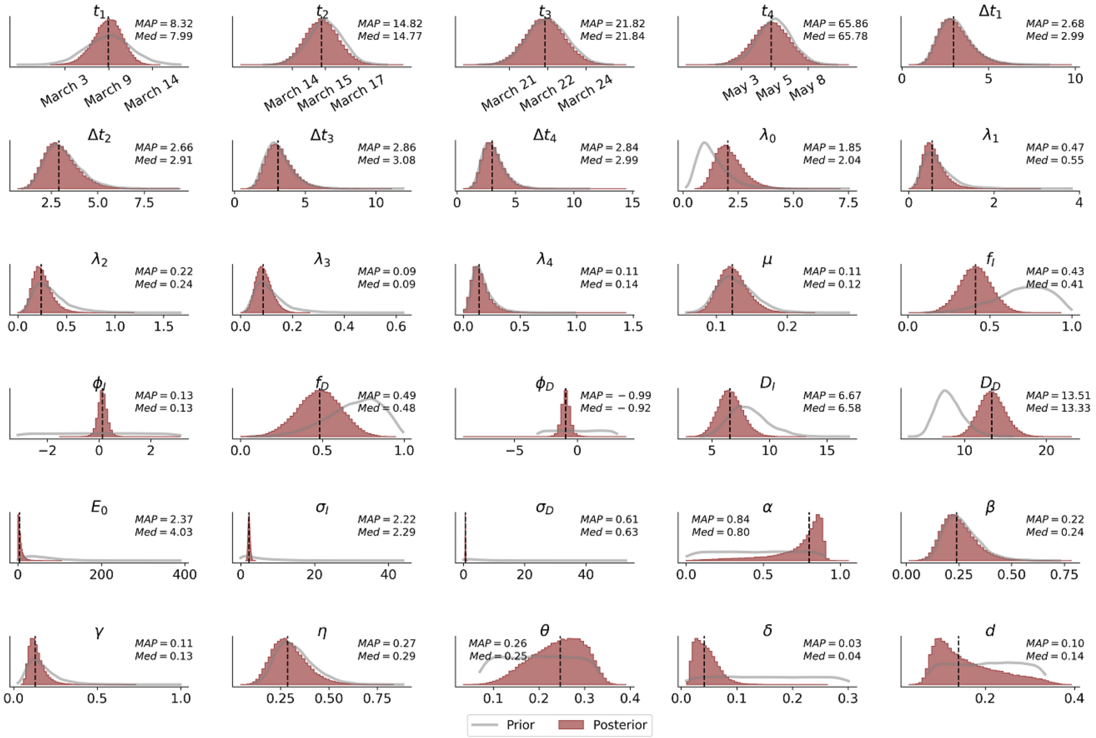

Fig S30. Marginal parameter posteriors from data available for the German federal state Schleswig-Holstein.

Thuringia

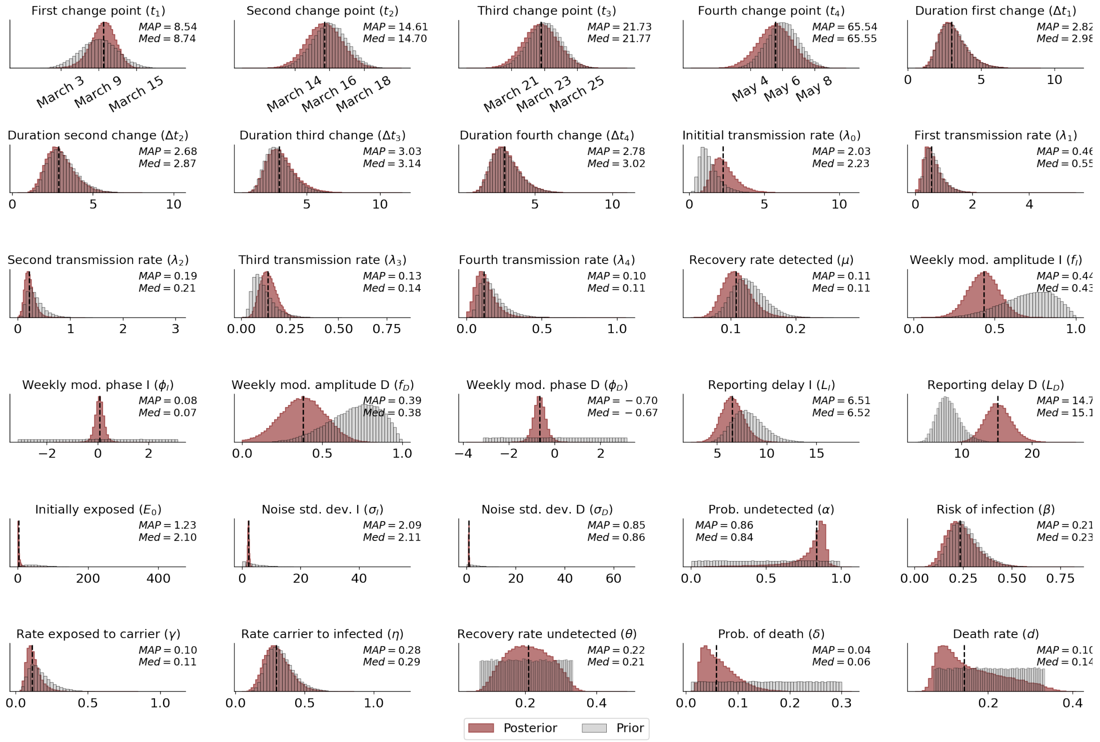

Fig S31. Marginal parameter posteriors from data available for the German federal state Thuringia.

References

1. Augeraud-Véron E, Sari N. Seasonal dynamics in an SIR epidemic system. Journal of mathematical biology. 2014;68(3):701–725.
